# Clozapine Reverses Dysfunction of Glutamatergic Neurons Derived From Clozapine-Responsive Schizophrenia Patients

**DOI:** 10.3389/fncel.2022.830757

**Published:** 2022-02-23

**Authors:** Hana Hribkova, Ondrej Svoboda, Elis Bartecku, Jana Zelinkova, Jana Horinkova, Lubica Lacinova, Martin Piskacek, Bretislav Lipovy, Ivo Provaznik, Joel C. Glover, Tomas Kasparek, Yuh-Man Sun

**Affiliations:** ^1^Department of Biology, Masaryk University, Brno, Czechia; ^2^Department of Biomedical Engineering, Faculty of Electrical Engineering and Communication, Brno University of Technology, Brno, Czechia; ^3^Department of Psychiatry, Faculty of Medicine and University Hospital Brno, Brno, Czechia; ^4^Center of Bioscience, Institute of Molecular Physiology and Genetics, Slovak Academy of Sciences, Bratislava, Slovakia; ^5^Department of Pathological Physiology, Masaryk University, Brno, Czechia; ^6^Department of Burns and Plastic Surgery, Faculty of Medicine and University Hospital Brno, Brno, Czechia; ^7^Department of Physiology, Faculty of Medicine, Masaryk University, Brno, Czechia; ^8^Department of Molecular Medicine, Institute of Basic Medical Sciences, University of Oslo, Oslo, Norway; ^9^Norwegian Center for Stem Cell Research, Department of Immunology and Transfusion Medicine, Oslo University Hospital, Oslo, Norway

**Keywords:** schizophrenia, clozapine, hiPSC, glutamate, neuron

## Abstract

The cellular pathology of schizophrenia and the potential of antipsychotics to target underlying neuronal dysfunctions are still largely unknown. We employed glutamatergic neurons derived from induced pluripotent stem cells (iPSC) obtained from schizophrenia patients with known histories of response to clozapine and healthy controls to decipher the mechanisms of action of clozapine, spanning from molecular (transcriptomic profiling) and cellular (electrophysiology) levels to observed clinical effects in living patients. Glutamatergic neurons derived from schizophrenia patients exhibited deficits in intrinsic electrophysiological properties, synaptic function and network activity. Deficits in K^+^ and Na^+^ currents, network behavior, and glutamatergic synaptic signaling were restored by clozapine treatment, but only in neurons from clozapine-responsive patients. Moreover, neurons from clozapine-responsive patients exhibited a reciprocal dysregulation of gene expression, particularly related to glutamatergic and downstream signaling, which was reversed by *c*lozapine treatment. Only neurons from clozapine responders showed return to normal function and transcriptomic profile. Our results underscore the importance of K^+^ and Na^+^ channels and glutamatergic synaptic signaling in the pathogenesis of schizophrenia and demonstrate that clozapine might act by normalizing perturbances in this signaling pathway. To our knowledge this is the first study to demonstrate that schizophrenia iPSC-derived neurons exhibit a response phenotype correlated with clinical response to an antipsychotic. This opens a new avenue in the search for an effective treatment agent tailored to the needs of individual patients.

## Introduction

Clozapine is one of the most effective antipsychotic agents (Meltzer, 2013; Okhuijsen-Pfeifer et al., 2018). About 30% of treatment-resistant schizophrenia patients receive clozapine (Farooq and Taylor, 2011). Nevertheless, some SCZ patients do not respond well to clozapine (Siskind et al., 2017) and it is hard to find an effective and safe treatment for this subgroup of patients. Clozapine resistance therefore poses a significant clinical challenge. Unfortunately, the processes behind clozapine non-response are unknown, and the mechanisms underlying selective clinical effects of clozapine remain unclear (Meltzer, 1994; Lameh et al., 2007; Dziedzicka-Wasylewska et al., 2008; Lee et al., 2017; Nucifora et al., 2017).

Until recently, progress in understanding the treatment and pathology of schizophrenia was hindered by the inability to study ongoing disease processes in human pathological tissue. Animal, human post-mortem and imaging studies indicate pathology in several brain cell types (Falk et al., 2016; van Erp et al., 2018). Pyramidal glutamatergic neurons—principal elements of cortical microcircuits—have reduced soma sizes and dendritic arbors (Harrison, 2000; Glausier and Lewis, 2013; van Erp et al., 2018). Moreover, changes in GABAergic interneurons lead to perturbations of coordinated activity in cortical microcircuits, and, consequently, a reduced ability to integrate inputs from other cortical areas, with subsequent abnormalities in cortico-cortical interactions (Garey, 2010; Gonzalez-Burgos and Lewis, 2012; Harrison, 2015; Datta and Arnsten, 2018).

Evidence from experiments with animals and non-pathological human or animal cell lines converges on the importance of glutamatergic signaling in the mechanism of clozapine action, together with induction of neuroplasticity and suppression of pro-inflammatory activity (Arvanov et al., 1997; Vallejo-Illarramendi et al., 2005; Tanahashi et al., 2012). Clozapine enhances NMDA receptor-mediated activation of cortical pyramidal neurons (Arvanov et al., 1997), and increases NMDA receptor-mediated glutamate output (Tanahashi et al., 2012). At the level of neuronal function, clozapine increases the firing rates of several neuronal types (Gessa et al., 2000; Schwieler and Erhardt, 2003; Nilsson et al., 2005; Schwieler et al., 2008). The mechanism of action is likely to be more complex than only activation of neurons. For example, Homayoun (Homayoun and Moghaddam, 2007) demonstrated differential effects on the basal activity of rat neurons and the suppression of MK801-induced neuronal hyperactivity in an acute pharmacological model of schizophrenia. Clozapine also increases the expression of synaptophysin (Bringas et al., 2012) and postsynaptic markers, and increases dendritic spine numbers and postsynaptic densities in rat hippocampal cell cultures (Critchlow et al., 2006), suggesting an induction of synaptic remodeling. This is supported by increased expression of cytoskeleton proteins and enhanced synaptogenesis in rat cortex after clozapine exposure (Kedracka-Krok et al., 2015; Chen et al., 2016). Important evidence comes from an animal model of schizophrenia—neonatal lesion of the ventral hippocampus—in which reconstitution of dendritic arbors and synaptogenesis in frontal cortex and nucleus accumbens were observed after clozapine treatment (Bringas et al., 2012). Whether any of these effects pertain to the treatment of human schizophrenic patients has yet to be determined, however.

The advent of induced pluripotent stem cell (iPSC) technology, permitting the generation of schizophrenic patient-specific neurons, has created new avenues for research into pathogenetic mechanisms. Brennand et al. (2011) used this approach to demonstrate that neurons generated from schizophrenia patients exhibit features predicted from post-mortem studies and showed that administration of lurasidone ameliorates disease-related phenotypes. Here, we have employed human iPSC-derived glutamatergic neurons to elucidate the molecular and cellular mechanisms that underlie clozapine-mediated effects in patients with different clinical response to clozapine.

## Materials and Methods

Details on Methods can be found in the Supplementary Material.

### Subjects

We have enrolled eight subjects [two healthy controls, three clozapine-responsive and three clozapine-resistant schizophrenia (SCZ) patients] into the current study. The inclusion criteria were: diagnosis of schizophrenia, prior or current treatment with clozapine, male gender, age between 20 and 60 years. General excluding criteria were substance abuse (allowing for caffeine and nicotine), somatic disorders affecting brain structure and function, another psychiatric diagnosis, IQ < 70, and inability to sign written consent. Two board-certified psychiatrists verified the diagnosis and assessed symptoms at the time of the recruitment by the M.I.N.I. structured interview (Sheehan et al., 1998). Other sources of data were archived patient documentation and an interview with a structure analogous to CASH-structured (The comprehensive assessment of symptoms and history) interview (Andreasen et al., 1992).

Two healthy adult male volunteers from the local community, from the same ethnic and age group as the patients, were asked to participate. They were recruited at the same time as the six SCZ patients.

All subjects signed the informed consent. The protocol of the study and informed consent form were approved by the local Ethical committees of the University hospital Brno and Faculty of Medicine, Masaryk University.

Clinical variables included symptom changes in relation to the initiation of clozapine treatment, pharmacological history, and clozapine response category: clozapine responsive (CLZ-R) and resistant (hereafter referred to as clozapine non-responsive, CLZ-NR) patients.

### Assessment of the Presence of Major Symptom Groups

Time-series of symptom changes in relation to the initiation of clozapine treatment and its long-term course were constructed. Symptoms were categorized as positive (delusions, hallucinations or formal thought disorder) and negative symptom groups (lack of motivation, anhedonia, lack of emotions, and poverty of speech).

### Pharmacological Patient History

First, time-series of medication changes were constructed and then compared to the time series of symptom changes. This algorithm permitted correlation of the effects of individual drugs and their combinations to different symptom groups. We identified the following treatment response patterns:

Full response—full resolution of the specific symptom group so that no further acute treatment was needed during the course of at least one episode.
Partial response—reduction of symptom severity so that no further acute treatment was needed during the course of at least one episode.
Ineffective—drug treatment with normally sufficient dose for normally sufficient time did not significantly ameliorate the symptom group.

### Clozapine Response

CLZ-R criteria: (1) the patient had to be treated with clozapine but no other medication aimed at symptoms of schizophrenia; (2) during the course of the disorder, the patient had to show either full effect of clozapine on major schizophrenic symptom groups (positive and negative symptoms) or a significant partial but sustained effect of clozapine. CLZ-NR criteria: (1) the patient had to be treated either with a combination of clozapine and another antipsychotic drug or with another antipsychotic medication alone; (2) during the course of the disorder, the patient had to show either no effect of clozapine or its combination with another medication on major schizophrenia symptoms, or a relapse of symptoms during the clozapine treatment that necessitated acute treatment.

To assess the neurocognitive status and the level of functioning of the patients we used Neurological Evaluation Scale (Buchanan and Heinrichs, 1989), Personal and Social Performance scale (Morosini et al., 2000) and neurocognitive battery (Kern et al., 2008) at the time of the biopsy for fibroblast harvest.

### Neuronal Differentiation

Eight hiPSC lines were generated from human dermal fibroblasts (for details of the protocol see Akkouh et al., 2021). Control and schizophrenia hiPSCs were harvested manually or dissociated with UltraPure™ EDTA and plated onto Matrigel-coated plates with differentiation media (for differentiation factors and the sequence of their application see Supplementary Material). Neural rosettes appeared at 8–20 days of differentiation, were harvested manually, and transferred to new plates. Neurons for experiments were derived from 2–3 clones from each iPSC line. The iPSC lines used to derive neurons were restricted to passage numbers 15–35 by the same scientist.

### Calcium Imaging

Neurons derived from control and SCZ iPSC lines were manually cut out from 90-day differentiation cultures, and seeded into separate μ-Slide 8 Well ibiTreat (Ibidi, 80826) where they settled, attached and were maintained in N2B27 medium until recording. Calcium influx was detected in non-phenol red N2B27 medium after staining the cells with 3 μM Fluo-4-AM ester (Invitrogen, F-14201) administered 30 min prior. After washing off the calcium indicator, cells were time-lapse filmed in the absence and presence of 10 mM glutamate (Gibco, 25030081) for 1 and 2.5 h, respectively. Twenty-seven regions of interest from each well were chosen for filming with an acquisition time of 1 image/2 min, after which the percentage of calcium-pulsing neurons was assessed and expressed as the number of neurons exhibiting repetitive calcium transients, expressed as a fold difference normalized to control neurons or neurons without exposure to glutamate or clozapine (in clozapine experiments, see below).

### Electrophysiology

Cell capacitance, resting membrane potentials, evoked action potentials, sodium and potassium currents, as well as glutamate-evoked EPSCs and NMDA receptor-mediated currents were recorded from glutamatergic neurons harvested at day 90. Whole-cell recordings were performed using electrodes (10–14 MΩ) pulled from borosilicate glass pipettes with filaments (BF150-86-10, Sutter Instruments) under an upright Nikon FN-S2N microscope. Signals were amplified with a two-channel patch clamp amplifier (EPC 10/2 USB, HEKA Elektronik), band pass filtered (2.9 kHz to 10 kHz) and digitized at a sampling frequency of 24 kHz. Cells were kept at 32°C during the experiments. Data were acquired using Patchmaster v2x90.1 and analyzed with FitMaster (v2x90.2, HEKA Elektronik) and OriginPro 2016 Sr2 (b9.3.2.303, OriginLab Corporation). Individual junction potentials calculated in Clampex (Molecular Devices) for each internal/bath solution pair were as follows: sodium current 5.2 mV, potassium current 4.5 mV, calcium current 5.3 mV, RMP, AP and capacitance 11.2 mV. Measured membrane potentials were corrected using these values. Current amplitudes were normalized to cell capacitance.

Sodium currents were activated by 20 ms long depolarizing pulses from a holding potential (HP) of −90 mV to voltages between −90 to + 60 mV in + 5 mV increasing increments. Potassium currents were activated by 500 ms long depolarizing pulses from a HP of −90 mV to voltages between −80 to +80 mV in + 10 mV increasing increments. NMDA currents and NMDA-evoked EPSCs were recorded for several seconds to ensure that no spontaneous EPSCs occurred, then cells were puffed with 200 μM NMDA and recorded continued for another 2 min.

### Effect of Clozapine

In experiments investigating the effect of clozapine on neuronal activity (spontaneous and glutamate-induced), cultures were prepared as described above but pretreated with 1 μM clozapine for 2 weeks prior to electrophysiological analyses.

### Immunocytochemical Analysis

Immunocytochemistry was performed as described previously (Grabiec et al., 2016). Quantitative analysis of NMDA-R-immunopositive puncta was performed using ImageJ software. The mean of each sample was obtained from 3 separate experiments and data were presented as fold difference in synapse number in SCZ neurons relative to control neurons. For the list of antibodies used for immunocytochemistry see Supplementary Material.

### RNA Sequencing

Neurons were collected at around 70 days (w/o 2-week clozapine exposure), when cultures contained predominantly (85–90%) glutamatergic neurons. RNA sequencing and data analysis was performed at Novogene, Hong Kong. The RNAseq was performed in paired-end 75 bp reads and at 20 million reads per sample.

### Data Analyses

Neurons for experiments were derived from 2–3 clones from each iPSC line. The statistics in calcium imaging experiments were computed from means of each sample obtained from 3 separate experiments; each experiment was performed with 200 neurons from each sample. Group-level K + and Na + I/V curves, number of action potentials, amplitude of action potentials and spike-frequency adaptation were calculated from pooled data from 10 neurons each from 2 control, 3 CLZ-R, and 3 CLZ-NR cultures. Differences of glutamate induced EPSPs and NMDA receptor-mediated currents between cultures of SCZ neurons with and without clozapine pretreatment were calculated from data obtained from 15 neurons in each culture.

All group differences were analyzed by non-parametric Mann–Whitney *U* Test with the significance level set to *p* < 0.05.

RNAseq data were analyzed using bioinformatic tools. The clean reads were aligned to the reference genome with Tophat2 software^1^ (Kim et al., 2013). Isoform assembly and quantification was done using the Cufflinks software (Trapnell et al., 2010). The quantification of expression and analysis of differential transcripts was done using Cuffdiff tool^2^ (Trapnell et al., 2010) to determine Fragments Per Kilobase of transcript sequence per Millions base-pairs sequenced (FPKM), method of estimating gene expression levels (Trapnell et al., 2010).

The differential transcripts were visualized using the volcano plots to show the up- and down-regulated genes between samples. The threshold was set to *p* < 0.05. Venn diagrams were constructed to visualize common and sample-unique differential transcripts.

Next, we have used Gene ontology using GOseq R package (Young et al., 2010). The pathway enrichment analysis to detect differentially expressed gene assemblies that support individual biological processes was performed with the use of KEGG [Kyoto Encyclopedia of Genes and Genomes, (Kanehisa et al., 2008)] pathways using KOBAS tool (Mao et al., 2005). A geometric test was calculated to interrogate significantly enriched pathway based on background genes and differentially expressed genes list. Significance threshold was set to FDR ≤ 0.05 to select enriched pathways. Rich factor is the ratio between enriched candidate genes number (cg) and total annotated genes number in individual pathway (bg). The results are presented as scatter plots of KEGG enrichment with number of enriched genes, Rich factor and q-value (a multiple test adjusted *p*-value: the closer to 0 the more significantly enriched).

We have compared the transcriptomic patterns of glutamatergic neurons from SCZ and control cultures. Then we have compared the transcriptomic profiles of CLZ-R SCZ neurons with and without clozapine pretreatment to see whether and how clozapine pretreatment influences the set of dysregulated genes.

## Results

### Clinical Characteristics

**FIGURE 1:**
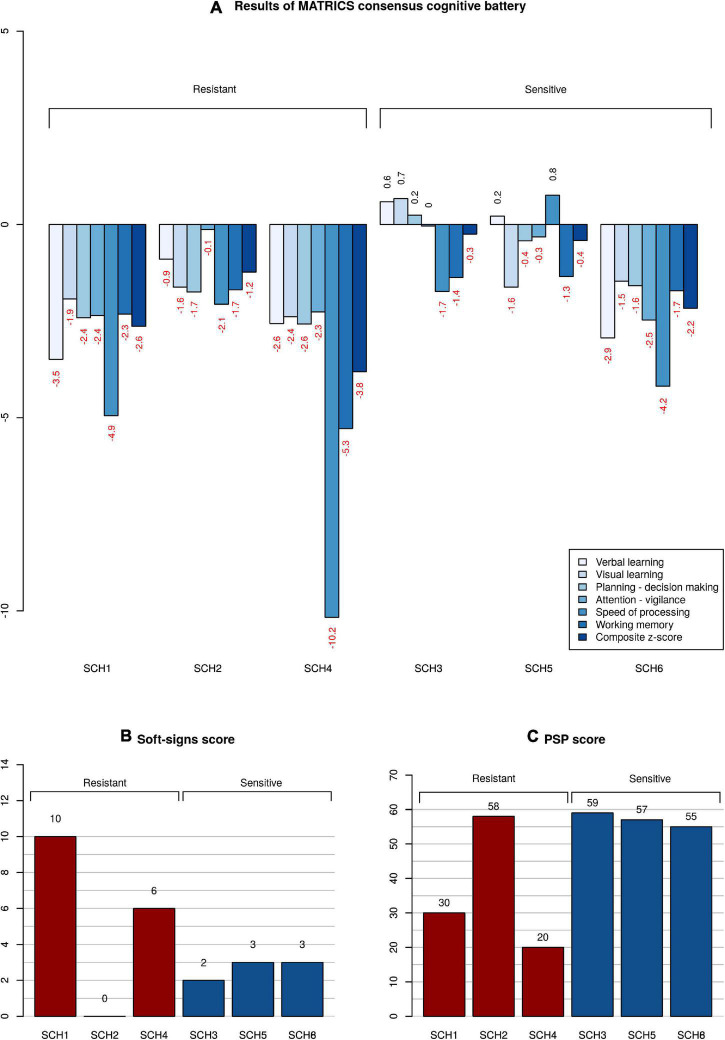
Neuropsychological, psychosocial, and neurological functioning of patients. **(A)** Z-scores of clozapine resistant and sensitive patients in comparison with a cohort of healthy subjects. Subtests of MATRICS battery are color-coded; the composite score calculated from all the subtests is the right-most column. **(B)** Magnitude of neurological soft signs expressed as a total Neurological evaluation scale score. The higher the score, the more severe the neurological abnormality. **(C)** Level of functioning given as a Personal and Social Performance scale score. The higher the score, the better the overall functioning.

The clinical and demographic information on subjects is given in Table 1. Clozapine treatment alone ameliorated the three principal syndromes of schizophrenia in CLZ-R patients (SCZ3, 5, 6), whereas CLZ-NR patients (SCZ1, 2, 4) exhibited inadequate responses to clozapine and required treatment with another drug. SCZ1 and SCZ4 in particular exhibited marked neurological abnormalities, poor social functioning, and severe cognitive deficits (Figure 1).

**TABLE 1:** Clinical characteristics of the samples.

			Symptoms before treatment					Symptom response to clozapine	
Subject	Group	Age	*P*	*N*	Illness duration	Years on clozapine	Medication at time of sampling	*P*	*N*
SCZ1	Resist.	41	Yes	Yes	12	5	CZL, HAL	Part./temp.	Part./temp.
SCZ2	Resist.	38	Yes	Yes	20	19	CLZ, RIS	Full/temp.	Ineff.
SCZ3	Resp.	31	Yes	Yes	3	3	CLZ	Full	Full/temp.
SCZ4	Resist.	39	Yes	Yes	13	10	CLZ, OLA, AMI, VAL	Part./temp.	Ineff.
SCZ5	Resp.	49	Yes	Yes	21	17	CLZ	Full	Part.
SCZ6	Resp.	38	Yes	Yes	12	10	CLZ	Part.	Part.
Mean		39.3			13.5	10.67			

### Cell Lines

**FIGURE 2:**
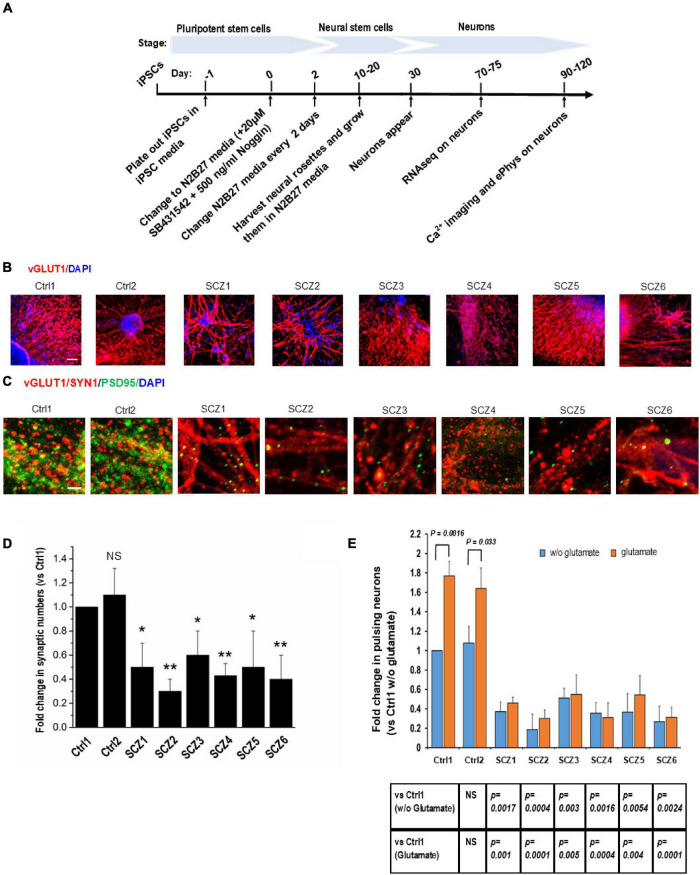
Cell line characteristics. **(A)** Timeline of the experiment. **(B)** Immunohistochemistry imaging of glutamatergic neurons derived from hiPSC lines (vGLUT1 antibody). **(C)** Immunohistochemistry imaging of postsynaptic densities of glutamatergic neurons (PSD95 antibody). **(D)** Number of postsynaptic densities of glutamatergic neurons. **(E)** Ca imaging, number of the ca transients and the effect of glutamate. CTRL, neurons from control subjects; SCZ, neurons from schizophrenia subjects. The symbols represent significance levels: *p < 0.05; **p < 0.001.

Neural differentiation using a standardized protocol (Figure 2A) generated primarily glutamatergic neurons appearing at 30 days (Figure 2B). By 90 days, neurons had elaborated synaptic contacts (Figures 2C,D) and exhibited spontaneous activity and responses to glutamate in the form of intracellular calcium transients (Figure 2E).

### Neuronal Activity

Fold change in the number of neurons with spontaneous recurrent calcium transients relative to a sample from a control subject was significantly lower in all SCZ cultures (40–70% fewer active neurons than in the control cultures, Figure 2E).

The response of neurons to glutamate was significantly larger in control neurons (56–77% increase in active neurons) compared to SCZ neurons (from 5% decrease to 18% increase in active neurons, Figure 2E).

**FIGURE 3:**
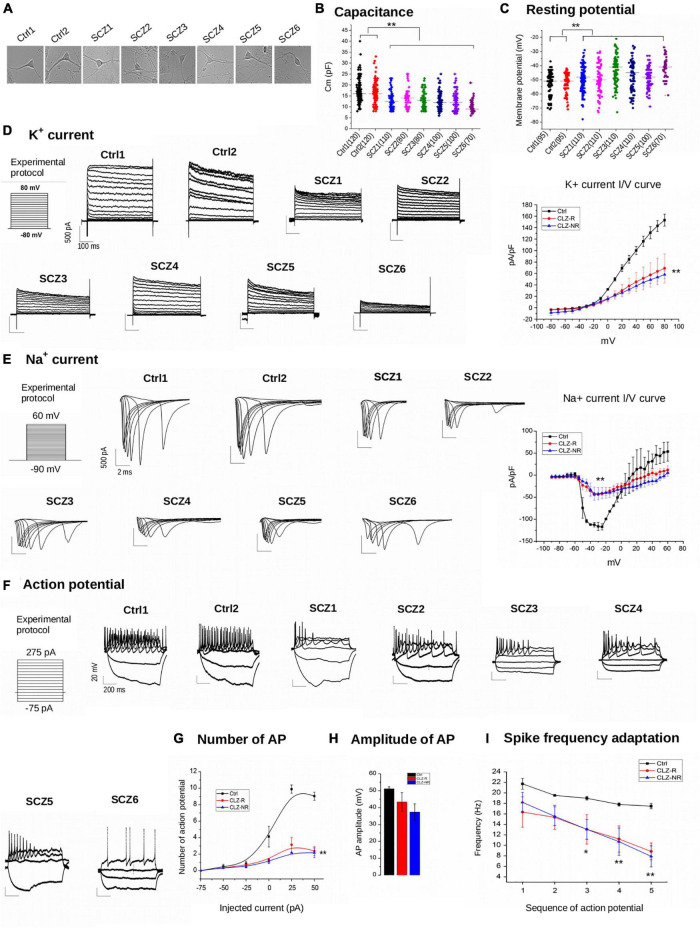
Electrophysiology. **(A)** Representative examples of samples. **(B)** Capacitance of individual cell lines. **(C)** Resting potential of individual neuronal lines. **(D)** K + current density charts of individual neuronal lines and aggregated K + current I/V curve. **(E)** Na + current density charts and aggregated Na + current I/V curve. **(F)** Action potential charts of individual neuronal lines. **(G)** Number of potentials in subgroups of neuronal lines. **(H)** Amplitude of action potentials in subgroups of neuronal lines. **(I)** Spike frequency adaptation in subgroups of neuronal lines. Ctrl, neurons from control subjects; CLZ-R, neurons from clozapine responsive schizophrenia patients; CLZ-NR, neurons from clozapine non-responsive schizophrenia patients. The symbols represent significance levels: *p < 0.05; **p < 0.001.

Schizophrenia neurons (Figure 3A) had about 25% lower capacitance than control neurons (12.7 ± 0.48 pF vs. 16.6 ± 0.46 pF, Figure 3B). Moreover, SCZ neurons had significantly less negative resting membrane potentials (−46.6 ± 0.9 mV vs. −51.9 ± 0.9 mV, Figure 3C). We found no differences in capacitance or membrane potential between CLZ-R and CLZ-NR SCZ neurons.

The current density of voltage-gated K + currents was significantly lower in SCZ neurons (Figure 3D). Inward Na + currents also had lower current densities in SCZ neurons (Figure 3E), indicating diminished excitability. We found no differences in current densities between CLZ-R and CLZ-NR SCZ neurons.

Schizophrenia neurons fired fewer than a third of the number of action potentials compared to control neurons to maintained current injection, with the highest difference evident at an injected current of 50 mA (9.0 ± 0.5 action potentials in control neurons vs. 2.4 ± 0.5 and 2.2 ± 0.6 in CLZ-R and CLZ-NR SCZ neurons, respectively, Figures 3F,G). The action potential amplitudes were lower in SCZ neurons (51.2 ± 1.6 mV in control neurons vs. 43.3 ± 9.7 mV and 37.3 ± 8.5 mV in CLZ-R and CLZ-NR SCZ neurons, respectively, Figure 3H). Spike frequency adaptation (SFA—decrement in the instantaneous frequency of APs) was augmented in SCZ neurons (Figure 3I).

### Immunocytochemistry of NMDA-R and PSD95

Immunocytochemistry demonstrated a lower density of glutamatergic synapses, as revealed by co-localization of NMDA receptor (NMDA-R) and PSD95 immunostaining (Figures 2C,D) in SCZ cultures. The fold difference in synapse number in SCZ cultures relative to control cultures ranged from 0.3 to 0.6. This correlated with a reduction in NMDA-R expression as assessed by RNAseq (see below).

### Effect of Clozapine Pretreatment on Neuronal Activity

**FIGURE 4:**
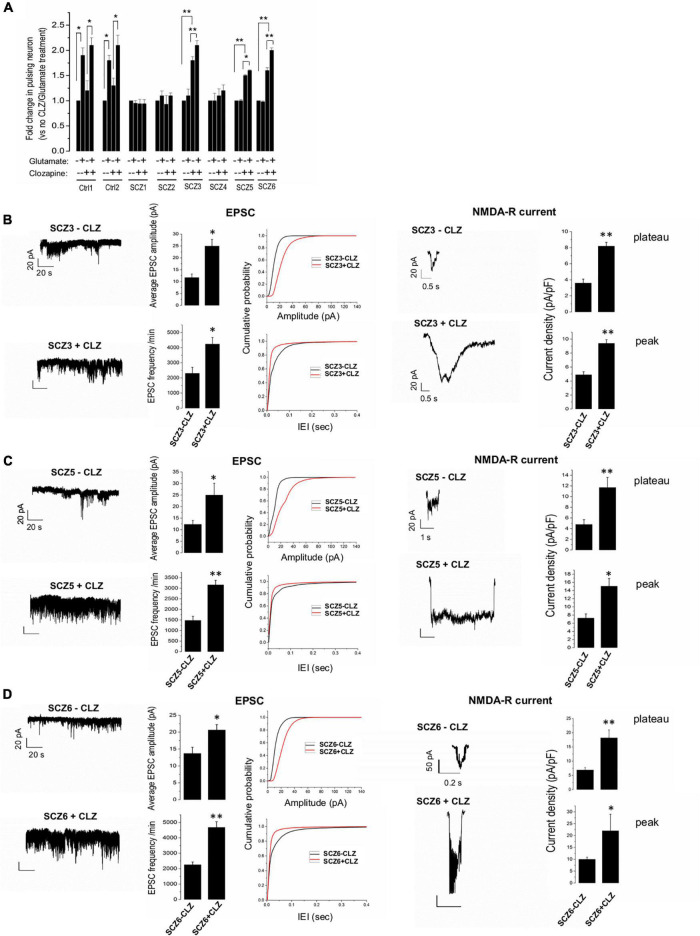
EPSPs, NMDA currents, and the effect of clozapine. **(A)** Ca imaging with and without glutamate and clozapine exposure. **(B–D)** EPSC and NMDA currents with and without clozapine pretreatment in neuronal lines from clozapine responsive patients. The symbols represent significance levels: *p < 0.05; **p < 0.001.

In general, SCZ neurons showed much less glutamate-induced activity than control neurons in the absence of clozapine pretreatment (Figure 4A). Clozapine pretreatment, however, markedly boosted both spontaneous and glutamate-induced activity in both control neurons and CLZ-R SCZ neurons, but not CLZ-NR SCZ neuron (Figure 4A). In CLZ-R SCZ neurons (SCZ 3, 5, 6), clozapine pretreatment enhanced spontaneous activity by 50–80%, and glutamate-induced activity by 60–210%. Activity changes in CLZ-NR SCZ neurons (SCZ 1, 2, 4) remained within the range of 10–20%.

Clozapine pretreated CLZ-R SCZ neurons consistently exhibited a significant twofold increase in EPSC amplitude and frequency, and NMDA-R-mediated plateau and peak current amplitudes (Figures 4B–D). Clozapine pretreatment had no significant effect on these features in CLZ-NR SCZ neurons (Supplementary Figure 1).

### Transcriptomic Analysis

RNAseq provided more than 40 million reads from each sample. The proportion of total mapped reads was above 85%, and of protein-coding RNA was above 80% in all samples.

### CLZ-R vs. CLZ-NR

**FIGURE 5:**
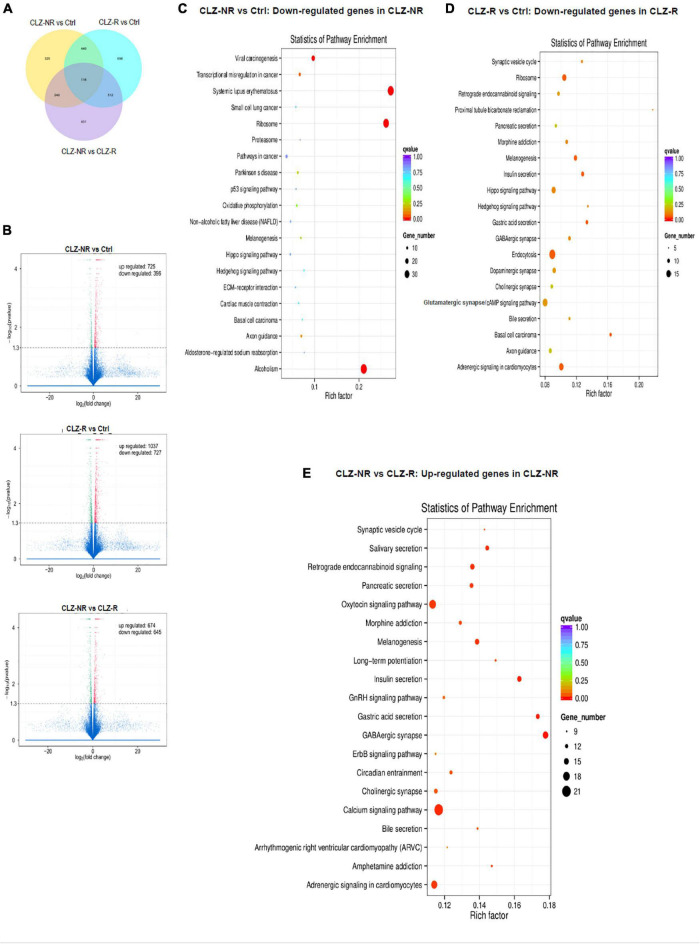
RNAseq profiles in clozapine responsive and resistant subjects. The results of transcriptomic profiling. **(A)** The number of gene expression changes. **(B)** Volcano plots comparing gene expression profile differences between clozapine responsive (CLZ-R), clozapine resistant (CLZ-R) and healthy controls (Ctrl). Pathway enrichment analysis comparing clozapine resistant patients with controls **(C)**, clozapine responsive patients with controls **(D)**, and clozapine resistant and responsive patients **(E)**.

To decipher the molecular basis of patient-selective responses to clozapine treatment, we employed transcriptomic profiling (Table 2 and Figure 5). Bioinformatics analysis revealed a distinctly different transcriptomic pattern in neurons derived from CLZ-R and CLZ-NR patients. In particular, many genes associated with retrograde endocannabinoid signaling, morphine addiction, and GABAergic, dopaminergic, cholinergic and glutamatergic synapses were all down-regulated in CLZ-R SCZ neurons, but not CLZ-NR SCZ neurons, relative to control neurons (Figures 5C,D, Supplementary Figures 2A,B, and Supplementary Table 2). Strikingly, genes in many of these pathways (except dopaminergic and glutamatergic signaling) were significantly up-regulated in CLZ-NR compared to CLZ-R SCZ neurons (Figure 5E, Table 2, and Supplementary Figure 2C). Moreover, expression of various voltage-gated K ^+^ and Na ^+^ channel genes was down-regulated in CLZ-R SCZ neurons (Supplementary Table 1), mirroring their aberrant electrophysiological properties.

**TABLE 2:** RNAseq pathway enrichment results.

SCZ-NR vs. Ctrl	SCZ-R vs. Ctrl	SCZ-R (+CLZ) vs. SCZ-R (−CLZ)	SCZ-NR vs. Ctrl	SCZ-R vs. Ctrl	SCZ-R (+CLZ) vs. SCZ-R (−CLZ)	SCZ-NR vs. SCZ-R	SCZ-NR vs. SCZ-R
**Down-regulated genes in SCZ-NR**	**Down-regulated genes in SCZ-R**	**Up-regulated genes in SCZ-R (+CLZ)**	**Up-regulated genes in SCZ-NR**	**Up-regulated genes in SCZ-R**	**Down-regulated genes in SCZ-R (+CLZ)**	**Up-regulated genes in SCZ-NR**	**Down-regulated genes in SCZ-NR**
1. Viral carcinogenesis	1. Synaptic vesicle cycle	1. VEGF signalling pathway	1. TGF-beta signalling pathway	2. TGF-beta signalling pathway	4. TGF-beta signalling pathway	1. Synaptic vesicle cycle	1. Wnt signalling pathway
2. Transcriptional misregulation in cancer	2. Pancreatic secretion	3. Rap1 signalling pathway	2. Renal cell carcinoma	3. Signalling pathways regulating pluripotency of stem cells	1. Viral carcinogenesis	2. Salivary secretion	2. Viral carcinogenesis
3. Systemic lupus erythematosus	3. Retrograde endocannabinoid signalling	2. Retrograde endocannabinoid signalling	3. Regulation of actin cytoskeleton	4. Regulation of actin cytoskeleton	2. Transcriptional misregulation in cancer	3. Retrograde endocannabinoid signalling	3. Tyrosine metabolism
4. Small cell lung cancer	4. Proximal tubule bicarbonate reclamation	4. Proximal tubule bicarbonate reclamation	4. Proteoglycans in cancer	5. Proteoglycans in cancer	5. Systemic lupus erythematosus	4. Pancreatic secretion	4. Transcriptional misregulation in cancer
5. Ribosome	2. Ribosome	5. Oxytocin signalling pathway	5. Prostate cancer	6. Platelet activation	6. Small cell lung cancer	5. Oxytocin signalling pathway	5. TGF-beta signalling pathway
6. Proteasome	6. Morphine addiction	7. Morphine addiction	6. PI3K-Akt signalling pathway	7. PI3K-Akt signalling pathway	3. Thyroid cancer	6. Morphine addiction	6. Systemic lupus erythematosus
7. Pathways in cancer	12. GABAergic synapse	13. GABAergic synapse	7. Pathways in cancer	8. Pathways in cancer	7. Pathways in cancer	7. Melanogenesis	7. Signalling pathways regulating pluripotency of stem cells
8. Parkinson’s disease	8. Insulin secretion	9. Insulin secretion	8. Malaria	1. Tight junction	8. Mismatch repair	8. Long-term potentiation	8. Pentose phosphate pathway
9. p53 signalling pathway	14. Dopaminergic synapse	15. Dopaminergic synapse	9. Hypertrophic cardiomyopathy (HCM)	9. Hypertrophic cardiomyopathy (HCM)	10. Hypertrophic cardiomyopathy (HCM)	9. Insulin secretion	9. Pathways in cancer
10. Oxidative phosphorylation	15. Cholinergic synapse	8. Long-term potentiation	10. HTLV-1 infection	10. HTLV-1 infection	11. HTLV-1 infection	10. GnRH signalling pathway	10. Melanogenesis
11. Non-alcoholic fatty liver disease (NAFLD)	11. Gastric acid secretion	12. Gastric acid secretion	11. Glycosphingolipid biosynthesis-lacto and neolacto series	11. Hippo signalling pathway	12. Hippo signalling pathway	11. Gastric acid secretion	11. Hypertrophic cardiomyopathy
12. Melanogenesis	7. Melanogenesis	6. Nicotine addiction	12. Gastric acid secretion	17. Complement and coagulaion cascades	9. Melanogenesis	12. GABAergic synapse	12. HTLV-1 infection
13. Hippo signalling pathway	9. Hippo signalling pathway	16. Cocaine addiction	13. Glioma	18. Cell cycle	18. Cell cycle	13. ErbB signalling pathway	13. Hippo signalling pathway
14. Hedgehog signalling pathway	10. Hedgehog signalling pathway	10. HIF-1 signalling pathway	14. Gap junction	14. DNA replication	16. DNA replication	14. Circadian entrainment	14. Falconic anemia pathway
15. ECM-receptor interaction	13. Endocytosis	14. Endocytosis	15. Focal adhesion	12. Focal adhesion	13. Focal adhesion	15. Cholinergic synapse	15. ECM-receptor interaction
16. Cardiac muscle contraction	16. Glutamatergic synapses/cAMP signalling pathway	11. Glutamatergic synapses	16. ECM-receptor interaction	13. ECM-receptor interaction	14. ECM-receptor interaction	16. Calcium signalling pathway	16. Cell cycle
17. Basal cell carcinoma	18. Basal cell carcinoma	17. Circadian entrainment	17. Dorso-ventral axis formation	16. Cytokine-cytokine receptor interaction	17. Colorectal cancer	17. Bile secretion	17. Basal cell carcinoma
18. Axon guidance	19. Axon guidance	18. Axon guidance	18. Dilated cardiomyopathy	15. Dilated cardiomyopathy	16. Dilated cardiomyopathy	18. Arrhythmogenic right ventricular cardiomyopathy (ARVC)	18. DNA replication
19. Aldosterone-regulated sodium reabsorption	17. Bile Secretion	19. Amphetamine addiction	19. Arrhythmogenic right ventricular cardiomyopathy (ARVC)	19. Arrhythmogenic right ventricular cardiomyopathy (ARVC)	19. Arrhythmogenic right ventricular cardiomyopathy (ARVC)	19. Amphetamine addiction	19. Arrhythmogenic right ventricular cardiomyopathy (ARVC)
20. Alcoholism	20. Adrenergic signaling in cardiomyocytes	20. ABC transporters	20. Amoebiasis	20. Amoebiasis	20. Alcoholism	20. Adrenergic signaling in cardiomyocytes	20. Alcoholism

### CLZ Pretreatment

**FIGURE 6:**
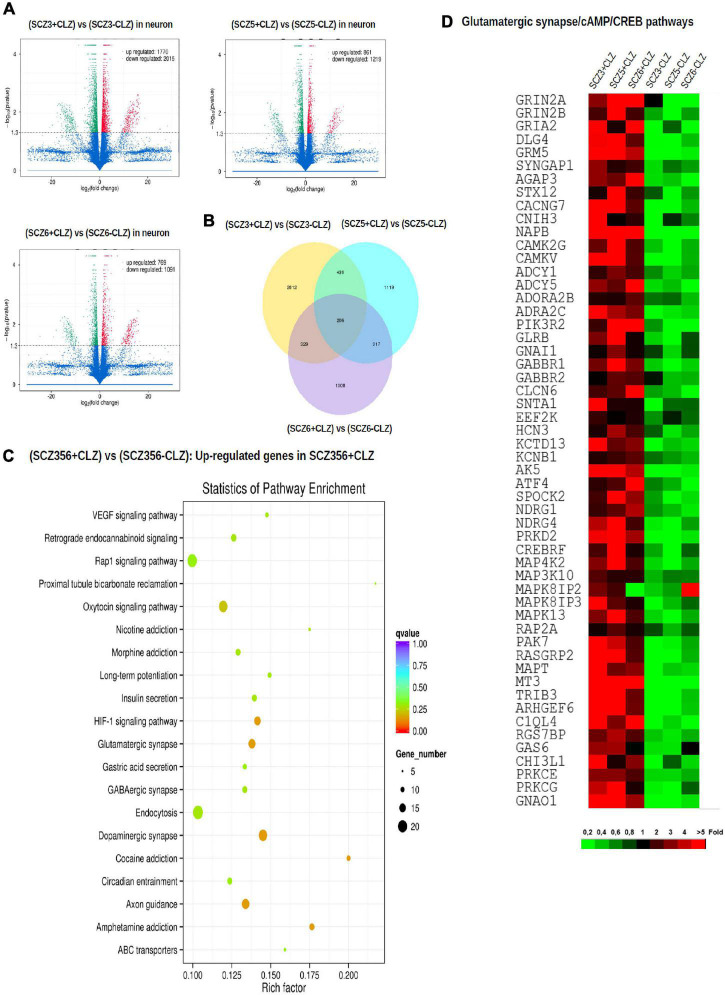
Gene expression after clozapine pretreatment. The results of transcriptomic profiling. **(A)** Volcano plots comparing the number of differentially expressed genes in individual clozapine responsive patients. **(B)** The number of genes with different expression profile in individual clozapine responsive patients. **(C)** Pathway enrichment analysis showing up-regulated genes after clozapine pretreatment. **(D)** Gene expression of glutamatergic, CREB, and cAMP pathways in cells from individual clozapine responsive patients before and after clozapine pretreatment. The magnitude of the expression is color-coded.

To pinpoint pathways through which clozapine acts, we made an additional transcriptomic assessment of CLZ-R SCZ neurons pretreated with clozapine for 2 weeks (Figure 6). Clozapine enhanced the expression of specific Na ^+^ and K ^+^ channel and transporter genes (Supplementary Table 2), reversing their down-regulation (Supplementary Table 1), and suggesting the associated electrophysiological phenotypes as targets of clozapine action. We found further that other gene sets that were down-regulated in CLZ-R SCZ neurons were up-regulated by clozapine pretreatment, including those involved in retrograde endocannabinoid signaling, morphine addiction, and GABAergic, dopaminergic, and glutamatergic synapses (Figure 6C, Supplementary Figure 3, and Table 2). In particular, clozapine pretreatment enhanced expression of numerous genes associated with glutamatergic signaling, including the NMDA-Rs GRIN2A and GRIN2B (Figure 6D). Clozapine up-regulated other genes in CLZ-R SCZ neurons, too, particularly genes associated with long-term potentiation, axon guidance, and nicotine, cocaine and amphetamine addiction (Figure 6C and Table 2). These results delineate and underscore the pleiotropic effects of clozapine at the level of gene expression.

## Discussion

Using hiPSC technology we have documented differences in neuronal activity, electrophysiological properties, and transcriptomic profiles between cultures of glutamatergic neurons derived from schizophrenia patients and healthy controls. These included reduced spontaneous and glutamate-evoked activity, lower inward Na + and outward K + current densities (indicating diminished excitability), reduced EPSC amplitudes and frequencies (paralleled by a lower density of glutamatergic synapses as revealed by co-localization of NMDA-R and PSD95 immunostaining). SCZ neurons fired at lower frequencies and with generally smaller spike amplitudes, and had greater spike frequency adaptation, indicating that they are less likely to maintain sustained high frequency firing than control neurons. Moreover, we have demonstrated that pretreatment with clozapine normalizes the aberrant pattern of electrophysiological function and transcriptomic changes selectively in neurons from clozapine-responsive patients, mirroring the clinical effect of clozapine in schizophrenia subjects.

Our transcriptomic profiling showed that gene sets involved in several signaling pathways are down-regulated in CLZ-R SCZ neurons compared to control or CLZ-NR SCZ neurons. These include gene sets related to retrograde endocannabinoid signaling, morphine addiction, GABAergic synapses, long-term potentiation, dopaminergic synapses, glutamatergic synapses, and axon guidance (Table 2). The question might arise whether the observed transcriptomic changes reflect a non-specific effect, such as cell manipulation (e.g., reprogramming), rather than a disease-related effect. However, some of the transcriptomic changes, including the expression of genes involved in synaptic plasticity and synaptic transmission, have been observed even in fibroblasts from schizophrenia patients, prior to the reprogramming steps of the iPSC pipeline (Etemadikhah et al., 2020).

Growing evidence points to a complex but specific dysregulation in schizophrenia of expression in gene sets encompassing several major biological processes: cytoskeleton, axon guidance, neurotransmission and synaptic function, calcium homeostasis, energy metabolism, oxidative stress, immune system and inflammation (Nascimento and Martins-de-Souza, 2015; Arion et al., 2017). Similar complex patterns of gene expression dysregulation have been observed in layer III pyramidal neurons of schizophrenia patients (Enwright et al., 2018). This is corroborated by the complex pattern of differential gene expression we observed in neurons derived from SCZ patients.

The same gene sets that are down-regulated in CLZ-R SCZ neurons return toward normal levels of expression following clozapine pretreatment (Table 2), suggesting that clozapine has a restorative effect on gene expression. It is noteworthy that these gene sets are not down-regulated in CLZ-NR SCZ neurons, suggesting that clozapine would not have a similar restorative effect in CLZ-NR SCZ neurons. Although this was not tested here, we found that clozapine pretreatment did not improve the electrophysiological function of CLZ-NR SCZ neurons, suggesting an absence of ion channel and neurotransmitter receptor-related gene expression changes in response to clozapine. Therefore, we infer that clozapine normalizes the transcriptome and improves the electrophysiological function of CLZ-R SCZ neurons selectively. To our knowledge this is the first study to demonstrate that hiPSC-derived neurons from schizophrenic patients recapitulate a differential clinical response to an antipsychotic drug.

Our observation of facilitated NMDA-R-mediated synaptic transmission accords with previous reports of a similar effect on rat prefrontal cells (Arvanov et al., 1997; Ninan and Wang, 2003) and of clozapine restoration of NMDA-mediated signaling in animal cell lines after NMDA-R antagonist administration (Chen and Yang, 2002). We find that clozapine upregulates the expression of human NMDA-R subunit genes (e.g., GRIN2A and GRIN2B), which could underlie clozapine-induced augmentation of NMDA-R currents in SCZ neurons. Other studies in rats, however, have shown that clozapine increases the release of excitatory amino acids rather than increasing the number of NMDA-Rs (Wittmann et al., 2005; Bragina et al., 2006; Tanahashi et al., 2012), and causes a decrease in the number of NMDA-Rs in the CA1 and CA2 regions of the hippocampus (Krzystanek et al., 2015).

Our findings extend a growing body of evidence that aberrant Na ^+^ and K ^+^ channel expression contributes to the etiology of schizophrenia (Imbrici et al., 2013; Carroll et al., 2016). We find dysregulated expression not only as previously reported for the Na ^+^ channels SCN2A and SCN3A and the K ^+^ channels KCNB1 and ATP1B1, but also for additional channel types not previously implicated in schizophrenia (e.g., SLC4A4, SLC32A1, SLC13A4, SLC1A4, SLC6A1, KCNK10, KCNH8, KCTD2). Furthermore, we show that SCZ neurons exhibit abnormally high spike frequency adaptation (SFA). SFA has been suggested to be a key element in working memory (Lansner et al., 2013), which is typically impaired in SCZ patients. Our novel finding that clozapine up-regulates Na ^+^ and K ^+^ channel and transporter genes in SCZ neurons and increases network activity supports the hypothesis that the alleviation by clozapine of the three major SCZ symptoms might originate in part from its actions on intrinsic neuronal excitability.

Transcriptomic profiles differ between neuronal cell types from the same cortical area and layer—only a small fraction (approx 17%) of differentially expressed genes in PN and PV of the III cortical layer from post-mortem samples of schizophrenia subjects overlap (Enwright et al., 2018). Our findings are confined to glutamatergic neurons, which are likely to represent pyramidal neurons *in vivo*. Changes and adaptations in other neural cell types (interneurons, astroglia, microglia etc.) almost certainly contribute to schizophrenia and to the clinical effect of clozapine. Furthermore, cortical location matters as well (Collado-Torres et al., 2019; Perez et al., 2021). iPSC-derived glutamatergic neurons obviously cannot fully reflect the regional differences in local microenvironment extant in the intact brain. On the other hand, iPSC technology is well positioned to address another variable—developmental time—since this can be recapitulated *in vitro*, for example in brain organoids (Stachowiak et al., 2017). It appears that in schizophrenia developmental changes play a critical role (Smith et al., 2019).

The ability of antipsychotics to influence the transcription of genes in schizophrenia has been demonstrated before. Crespo-Faccoro (Crespo-Facorro et al., 2014) observed normalization of blood transcriptome profiles after antipsychotic treatment—at least in some differentially expressed genes. Here, we have shown that clozapine-induced changes in gene expression profile relate to functional changes in affected neurons—notably the normalization of glutamate, K, and Na related gene expression correlates with the normalization of the response to glutamate, facilitation of NMDA-R-mediated transmission, and K + and Na + current amplitudes. However, the observed pattern of clozapine-induced gene expression changes is much more complex and extends beyond processes involved in glutamatergic neuron function. Similarly, a case report of monozygotic twins discordant for clozapine response showed that the twins differed in the expression of cell adhesion molecules in neurons derived from iPSCs (Nakazawa et al., 2017). Although these authors did not perform any pharmacological experiments to link their findings to clinical responses to clozapine, they also suggest that biological differences between clinically-defined phenotypes are complex and likely extend beyond processes directly related to an effect of clozapine.

Although we observed similar characteristic features in the six independent SCZ neuronal cultures, the sample size is too low to make clinical predictions. We note that at the start of the experiment (2015), hIPSC technology was a very laborious, time-consuming and resource-demanding approach. The sample size we have used is comparable to state-of-the art experiments published up to the present day (Stachowiak et al., 2017; Stertz et al., 2021). Still, the results are robust at the level of individual neurons, and have sufficient statistical power to demonstrate clear differences between different neuronal cultures, and the pattern of changes with respect to clozapine treatment is striking.

## Conclusion

Clozapine improves spontaneous and glutamate-induced activity, and NMDA-related electrical activity in glutamatergic neurons derived from clozapine-responsive, but not clozapine-resistant, schizophrenia patients. This functional improvement is mirrored by the normalization of a complex, aberrant transcriptomic profile. The electrophysiological and transcriptomic data implicate abnormal expression of NMDA-Rs and Na + and K + channels in schizophrenia and clozapine treatment-related effects. Further research is needed to corroborate these results in larger sample sizes and to fully elucidate the mechanisms underlying clozapine effects and the nature of clozapine resistance. Nevertheless, our findings show that hiPSC-derived neurons from clinically defined schizophrenia patients reflect at the cellular level differences in clinical treatment response, and thus provide a potentially relevant model for studying the mechanisms of antipsychotic action and patient-specific treatment predictions.

## Data Availability Statement

The original contributions presented in the study are publicly available. This data can be found here: https://www.ebi.ac.uk/ena/browser/home, PRJEB50039.

## Ethics Statement

The studies involving human participants were reviewed and approved by Ethical Committee of the University hospital Brno and Ethical Committee of the Faculty of Medicine, Masaryk University. The patients/participants provided their written informed consent to participate in this study.

## Author Contributions

Y-MS contributed to conceptualizing the study, designed all experiments (basic science part), and supervised the experiments carried out by HH. OS and JZ performed and assessed the activity of NMDA-R using whole-cell patch recording, analyzed and interpreted all data (including basic science work and RNAseq data), prepared the figures, wrote the manuscript, and approved the final manuscript version. HH contributed to the generation of iPSC lines and the derivation of neurons and astrocytes, per-formed immunocytochemical analysis, wrote the “Materials and Methods” section (basic science part), and reviewed the manuscript. TK contributed to all clinical work, including the study design, supervision of the subject characterization, data analysis, interpretation, co-wrote the manuscript. OS carried out whole-cell recording of potassium currents, sodium currents, and action potentials, by whole-cell patch, and wrote the “Materials and Methods” section (electrophysiological part). EB contributed to the clinical evaluation of patients, analysis of clinical data, and manuscript proofreading. LL contributed to the design and supervision of electrophysiology experiments, taught Y-MS the analysis of electrophysiology data, interpreted and drafted the electrophysiology data, and reviewed and edited the manuscript. JG contributed to the data interpretation, initial drafting of the manuscript, manuscript revision, and approval of the final manuscript version. JZ performed all calcium imaging recording. JH contributed to the recruitment of patients, data collection, and clinical evaluation of patients. IP sponsored and supervised the work carried out by OS. BL obtained skin biopsies and evaluated patients. MP produced heat maps of RNAseq data. All authors contributed to the article and approved the submitted version.

## Conflict of Interest

The authors declare that the research was conducted in the absence of any commercial or financial relationships that could be construed as a potential conflict of interest.

## Publisher’s Note

All claims expressed in this article are solely those of the authors and do not necessarily represent those of their affiliated organizations, or those of the publisher, the editors and the reviewers. Any product that may be evaluated in this article, or claim that may be made by its manufacturer, is not guaranteed or endorsed by the publisher.

## Footnotes

1
http://tophat.cbcb.umd.edu/

2
http://cole-trapnell-lab.github.io/cufflinks/cuffdiff/index.html
