# Supplementary material for: Clozapine Reverses Dysfunction of Glutamatergic Neurons Derived From Clozapine-Responsive Schizophrenia Patients
Source: Front Cell Neurosci. 2022 Feb 23;16:830757. doi: 10.3389/fncel.2022.830757 (PMC8904748; doi:10.3389/fncel.2022.830757)

Supplementary material

Methods

Scales and psychometric tests

To assess the long-term states of the patients we used Neurological Evaluation Scale, Personal and Social Performance scale and neurocognitive battery at the time of the biopsy for fibroblast harvest.

Neurological soft signs using Neurological Evaluation Scale (Buchanan et al., 1989) to detect discrete sensory-motor disturbances that reflect a progressive course of the illness (Buchanan et al., 1989; Bachmann et al., 2014); NES is a structured scale presenting scores in four subscales (sensory integration, motor coordination, sequencing of complex motor acts and "others" reflecting the presence of ontologically old reflexes). It captures a wide range of signs within 26 items. Each item is rated on a scale of 0-_2 (0 = relatively normal, 1 = some disruption, 2 = major disruption). The higher the score, the greater the neurological impairment. To measure the severity of neurological impairment, the total score was used.

Personal and Social Performance scale (Morosini et al., 2000) to measure the general level of patient functioning in day-to-day activities and the impact of symptoms on daily life (Juckel et al., 2008; Suttajit et al., 2015); The PSP score consists of four sub-domains each with six anchor points: socially useful activities, personal and social relationships, self-care, disturbing and aggressive behaviour. The PSP score has a value between 1 (life threatening condition) and 100 (normal level of functioning). The higher the score, the closer is a patient to a normal level of functioning normal in the population of the same age and socio-economic status.

MATRICS Consensus Cognitive Battery (MCCB) to assess neurocognitive status. MCCB consists of 6 cognitive domains: speed of processing, attention/vigilance, working memory, verbal learning, visual learning, reasoning and problem solving (Kern et al., 2008). Due to lack of normative data for the Czech population, we derived z-scores from a reference group composed of 37 (13 females) healthy individuals with no history of psychiatric or neurological disorder, with mean age 32 (SD = 8.69) and mean duration of education 14.89 years (SD = 2.69).

Neuronal differentiation

Differentiation factors and sequence of their application: hiPSCs were plated onto Matrigel-coated plates in Essential 8™ Medium (Gibco, A1517001). The next day (called D0), the medium was replaced with N2B27 medium supplemented with 20 µM SB431542 (Tocris, 1614) and 500 ng/ml Noggin (R&D Systems, 3344-NG). The supplements were applied for another 2 days and removed at 4 days of differentiation. Cells were then maintained in N2B27 medium alone, which was changed every second day. Neural rosettes appeared at around 8-20 days of differentiation, were harvested manually, and transferred to new plates pre-coated with Mat-rigel/Fibronectin (Sigma, F1141) or PDL (Sigma, P7280)/Laminin (Sigma, L2020)/Fibronectin (Sigma, F1141). Medium was then changed every second day until 30 days of differentiation and then every 3 days thereafter.

Electrophysiology

Na and K currents: For measuring **sodium currents**, pipettes were filled with internal solution containing 130 mM CsCl, 2 mM MgCl2, 5.5 mM EGTA, and 10 mM HEPES, pH

adjusted to 7.2 with CsOH and the bath solution contained 2.7 mM CsCl, 135 mM NaCl, 1 mM Na2HPO4, 2.5 mM EGTA, 1 mM MgCl2, 10 mM glucose, and 10 mM HEPES, pH adjusted to 7.4 with NaOH. Currents were activated by 20 ms long depolarizing pulses from a holding potential (HP) of -90 mV to voltages between -90 to +60 mV in +5 mV increasing increments. For measuring **potassium currents**, the internal solution contained 130 mM KCl, 2 mM MgCl2, 5.5 mM EGTA, and 10 mM HEPES, pH adjusted to 7.2 using KOH and the bath solution con-tained 135 mM NaCl, 2.7 mM KCl, 1 mM Na2HPO4, 2.5 mM EGTA, 1 mM MgCl2, 10 mM glucose, 10 mM HEPES, and 1 µM TTX, pH adjusted to 7.4 using NaOH. Currents were activated by 500 ms long depolarizing pulses from a HP of -90 mV to voltages between -80 to +80 mV in +10 mV increasing in-crements.

Resting membrane potentials, **evoked action potentials** and **cell capacitance** were measured using an internal pipette solution containing 130 mM KCl, 0.5 mM CaCl2, 2 mM MgCl2, 5 mM EGTA, and 10 mM HEPES, pH adjusted to 7.2 using KOH and a bath solution containing 135 mM NaCl, 2.7 mM KCl, 1 mM Na2HPO4, 2.5 mM CaCl2, 1 mM MgCl2, 10 mM glucose, and 10 mM HEPES, pH adjusted to 7.4 with NaOH .

NMDA currents and NMDA-evoked EPSCs were recorded using an internal pipette solution containing 140 mM CsCH3SO3, 4 mM NaCl, 0.5 mM CaCl2, 5 mM EGTA, 10 mM HEPES, 0.5 mM Na3GTP, and 2 mM MgATP, pH adjusted to 7.3 with CsOH and a bath solution containing 145 mM NaCl, 3 mM CsCl, 2 mM CaCl2, 10 µM glycine, 1 µM TTX, 10 mM HEPES, and 10 mM D-glucose, pH adjusted to 7.35 with CsOH. During the recordings, current traces were run for several seconds to en-sure that no spontaneous EPSCs occurred, then cells were puffed with 200 µM NMDA and recorded continued for another 2 min.

Effect of clozapine

In experiments investigating the effect of clozapine on cell activity – intrinsic and NMDA responses - cultures of neurons were prepared as described above but pre-treated with 1 µM clozapine for 2 weeks prior.

Clozapine (SML2304) was made up to 10 mM with sterile water and then diluted into 0.5, 0.75, 1, 2, 3, and 5 µM with culture medium. Cell were tested with those concentrations and observed the reaction of cells (cell death, healthy shapes..etc.). We then have chosen 1µM for the experimental concentration. Clozapine was diluted with medium so control experimental groups were given medium only. The time point of administering clozapine was two weeks before collecting samples for analysis.

Immunocytochemical analysis

Immunocytochemistry was performed as described previously (Grabiec et al., 2016). Primary antibodies were SSEA-4 (Invitrogen, MA1-021, 1:200), Nanog (Invitrogen, PA1-097, 1:200), TRA-1-81 (Invitrogen, MA1-024, 1:200), Oct-3/4 (Santa Cruz, sc-8629, 1:300), vGLUT1 (Sigma, V0389, 1:500), GFAP (Santa-Cruz, sc-6170, 1:200), SYN (Invitrogen, PA1-1043, 1:500), PSD95 (Invitrogen, MA1-046, 1:500), and GluN1 as a proxy for NMDA-R (Sigma, SAB2500698, 1:500). Alexa Fluor secondary antibodies were A-21207, A-21202, and A-11058 (Invitrogen, 1:500), and 711-175-152 (Jackson ImmunoResearch, 1:500).

Data analyses

RNAseq

The clean reads were aligned to the reference genome with Tophat2 software (http://tophat.cbcb.umd.edu/) (Kim et al., 2013) – it proceeds with the mapping of the full-length reads to the exons and partial reads/fragments to two exons. Total mapped reads or fragments should be larger than 70% if proper reference genome is available and there is no contamination during the experimental procedure. Isoform assembly and quantification was

done using the Cufflinks software (Trapnell et al., 2010). The quantification of expression and analysis of differential transcripts was done using Cuffdiff tool (http://cole-trapnell-lab.github.io/cufflinks/cuffdiff/index.html) (Trapnell et al., 2010) to determine Fragments Per Kilobase of transcript sequence per Millions base-pairs sequenced (FPKM), method of estimating gene expression levels, which takes into account the effects of both sequencing depth and gene length on counting fragments (Trapnell, Cole, et al., 2010). Reproducibility measures were performed using the comparison of expression levels under different experimental conditions through FPKM box plot and density distribution. Moreover, the correlation of gene expression among samples was computed as Pearson correlation coefficients (R2) - the correlation coefficient close to 1 indicates high similarity of gene expression among samples.
Gene ontology analysis - we used the GOseq R package (Young et al, 2010), based on Wallenius non-central hyper-geometric distribution. The Wallenius distribution, compared to hyper-geometric distribution, has the feature that the probability of sampling from a population is different from sampling from another one by assessing the bias of gene length, which can calculate the probability of GO term enrichment more accurately.
Pathway enrichment analysis was performed with the use of KEGG (Kyoto Encyclopedia of Genes and Genomes) pathways using KOBAS tool (Mao et al., 1995). KEGG is a public pathway related database (Kanehisa,2008). Each KEGG Pathway is considered as a unit genes set. A geometric test was calculated to interrogate significantly enriched pathway based on background genes and differentially expressed genes list. Significance threshold was set to FDR $\leq$ 0.05 to select enriched pathways. We calculated number of enriched genes per pathway (cg) and compared them with the total number of pathway genes (bg). Rich factor is the ratio between enriched candidate genes number (cg) and total anotated genes number in individual pathway (bg). The higher the Rich factor is the heavier the enrichment. The results are presented as scatter plots of KEGG enrichment with number of enriched genes, Rich factor and q-value (a multiple test adjusted p-value: the closer to 0 the more significantly enriched).

Supplementary Table 1: Downregulation of Na and K channel genes in clozapine responsive subjects

List of down-regulated voltage-gated K+ and Na+ channel genes in CLZ-R neurons with corresponding results of the Pathway enrichment analysis: Magnitude of the change (Log2, fold change), uncorrected p-value, q-value, and gene description.

SCZ356 vs Ctrl12

Down regulated genes in SCZ356

Na^+ channels

Name of gene	Log2 (fold change)	p value	q value	Gene description
SLC4A4	-2.43645	0.02285	0.43339	Sodium Bicarbonate Cotransporter
SLC32A1	-1.51848	0.0145	0.34003	GABA Vesicular Transporter, Member 1
SLC13A4	-0.96674	0.0064	0.21255	Sodium/Sulphate Symporters, Member 4
SLC1A4	-1.0186	0.0002	0.01905	Glutamate/Neutral Amino Acid Transporter, Member 4
SLC17A8	-1.39028	0.0052	0.18676	Vesicular Glutamate Transporter, Member 8
SCN2A	-1.23504	0.023	0.4349	Voltage-Gated Sodium Channel Subunit Alpha Nav1.2
ATP1B1	-0.81836	0.0443	0.61074	ATPase Na+/K+ Transporting Subunit Beta 1
SCN3A	-1.30922	0.0093	0.26543	Voltage-Gated Sodium Channel Subunit Alpha Nav1.3
ATP1A2	-2.11555	5.00E-05	0.00612	ATPase Na+/K+ Transporting Subunit Alpha 2
ATP1A3	-0.688	0.0145	0.34003	ATPase Na+/K+ Transporting Subunit Alpha 3
SLC6A1	-0.73698	0.0468	0.62735	Sodium/Chloride-Dependent GABA Transporter 1
HCN4	-0.9956	0.01005	0.27754	Potassium/Sodium Hyperpolarization-Activated Cyclic Nucleotide-Gated Channel 4
SLC17A6	-0.7205	0.01555	0.35331	Vesicular Glutamate Transporter, Member 6

K^+ channels

Name of gene	Log2 (fold change)	p value	q value	Gene description
KCNK10	-1.38612	0.01015	0.27913	Outward Rectifying Potassium Channel Protein TREK-2
KCNB1	-0.6707	0.036	0.54987	Voltage-Gated Potassium Channel Subunit Kv2.1
KCNH8	-0.80098	0.0049	0.17997	Voltage-Gated Potassium Channel Subunit Kv12.1
KCTD2	-0.7488	0.0426	0.59894	Potassium Channel Tetramerization Domain-Containing Protein 2
ATP1B1	-0.81836	0.0443	0.61074	ATPase Na+/K+ Transporting Subunit Beta 1
KCNQ2	-0.63314	0.0368	0.55615	Voltage-Gated Potassium Channel Subunit Kv7.2
ATP1A2	-2.11555	5.00E-05	0.00612	ATPase Na+/K+ Transporting Subunit Alpha 2
ATP1A3	-0.688	0.0145	0.34003	ATPase Na+/K+ Transporting Subunit Alpha 3
TMEM38A	-1.08508	0.0243	0.44762	Trimeric Intracellular Cation Channel Type A
KCNG1	-1.30284	0.00015	0.01521	Voltage-Gated Potassium Channel Subunit Kv6.1
KCNF1	-1.67608	0.00145	0.08078	Voltage-Gated Potassium Channel Subunit Kv5.1
KCNJ4	-1.97765	5.00E-05	0.00612	Inward Rectifier K(+) Channel Kir2.3
HCN4	-0.9956	0.01005	0.27754	Potassium/Sodium Hyperpolarization-Activated Cyclic Nucleotide-Gated Channel 4

Supplementary Table 2: Up-regulation of Na and K channel genes after clozapine pretreatment

List of up-regulated voltage-gated K+ and Na+ channel genes in CLZ-R neurons after pretreatment with clozapine, with corresponding results of the Pathway enrichment analysis: Magnitude of the change (Log2, fold change), uncorrected p-value, q-value, and gene description.

SCZ356+CLZ vs SCZ356-CLZ

Up-regulated genes in SCZ356+CLZ

	log2 (foldchange)	p valve	q value	Na+/H+ exchanger
Na^+ channels				
SLC9A5	2.01373	0.04585	0.6211	Na+/H+ exchanger
SLC9A9	1.66046	0.0029	0.1283	Solute Carrier Family 9 Member A9
SLC4A4	0.8528	1.28295	0.0089	Na(+)/HCO3(-) Cotransporter
HCN3	0.93432	0.00265	0.12092	Hyperpolarization Activated Cyclic Nucleotide Gated Potassium Channel 3
SLC6A17	1.37969	0.0003	0.02598	Sodium:neurotransmitter symporter
SLC17A7	0.9043	0.00035	0.0292	Sodium-Dependent Inorganic Phosphate Cotransporter
SCN2A	0.95725	0.0441	0.60942	Voltage-Gated Sodium Channel Subunit Alpha Nav1.2
SLC6A6	0.75369	0.0287	0.48866	Sodium- And Chloride-Dependent Taurine Transporter
SLC6A1	1.8472	5.00E-05	0.00612	Sodium:neurotransmitter symporter
SLC38A3	0.87424	0.028	0.48233	System N1 Na+ And H+-Coupled Glutamine Transporter
SLC1A1	1.17348	0.00155	0.08448	Sodium-Dependent Glutamate/Aspartate Transporter 3
SLC6A11	2.49038	5.00E-05	0.00612	Sodium:neurotransmitter symporter
ATP1A3	0.71891	0.0041	0.16081	ATPase Na+/K+ Transporting Subunit Alpha 3
ATP1B2	0.82226	0.01055	0.28525	ATPase Na+/K+ Transporting Subunit Beta 2
SLC10A5	0.99379	0.03325	0.52791	Sodium/Bile Acid Cotransporter 5
K^+ channels				
KCNJ9	1.17267	0.0006	0.0434	Potassium Voltage-Gated Channel Subfamily J Member 9
KCNAB2	0.97666	0.04185	0.59374	Potassium Voltage-Gated Channel Subfamily A Regulatory Beta Subunit 2
KCNV1	2.17907	0.0001	0.01097	Potassium Voltage-Gated Channel Modifier Subfamily V Member 1
KCNB1	0.87719	0.00235	0.11157	Potassium Voltage-Gated Channel Subfamily B Member 1
KCTD16	0.60646	0.0368	0.55615	Potassium Channel Tetramerization Domain Containing 16
HCN3	0.93432	0.00265	0.12092	Hyperpolarization Activated Cyclic Nucleotide Gated Potassium Channel 3
KCNQ4	1.38145	0.0048	0.17766	Potassium Voltage-Gated Channel Subfamily Q Member 4
KCNK12	1.03873	0.00205	0.10182	Potassium Two Pore Domain Channel Subfamily K Member 12
KCTD13	0.96674	0.0064	0.21255	Potassium Channel Tetramerization Domain Containing 13
CNTNAP1	0.6498	0.01625	0.36188	Contactin Associated Protein 1
KCTD2	0.71298	0.03295	0.52537	Potassium Channel Tetramerization Domain Containing 2
KCNQ2	0.94575	0.00075	0.05104	Potassium Voltage-Gated Channel Subfamily Q Member 2
KCNJ8	1.07145	0.0132	0.32302	Inward Rectifier K(+) Channel Kir6.1
KCND1	1.22705	0.00265	0.12092	Potassium Voltage-Gated Channel Subfamily D Member 1
ATP1A3	0.71891	0.0041	0.16081	ATPase Na+/K+ Transporting Subunit Alpha 3
TMEM38A	1.12062	0.0096	0.27036	Transmembrane Protein 38A
KCNF1	1.12451	0.01715	0.3724	Voltage-Gated Potassium Channel Subunit Kv5.1
KCNIP2	1.21224	0.00855	0.25276	Potassium Voltage-Gated Channel Interacting Protein 2
KCNJ4	1.66354	0.0002	0.01905	Potassium Voltage-Gated Channel Subfamily J Member 4
ATP1B2	0.82226	0.01055	0.28525	ATPase Na+/K+ Transporting Subunit Beta 2
KCNA4	1.02662	0.01	0.27672	Potassium Voltage-Gated Channel Subfamily A Member 4
HCN4	0.69641	0.03955	0.57679	Hyperpolarization Activated Cyclic Nucleotide Gated potassium Channel 4

Supplementary Figure 1: Effect of clozapine on EPSC amplitude and frequency in neurons from clozapine-resistant patients

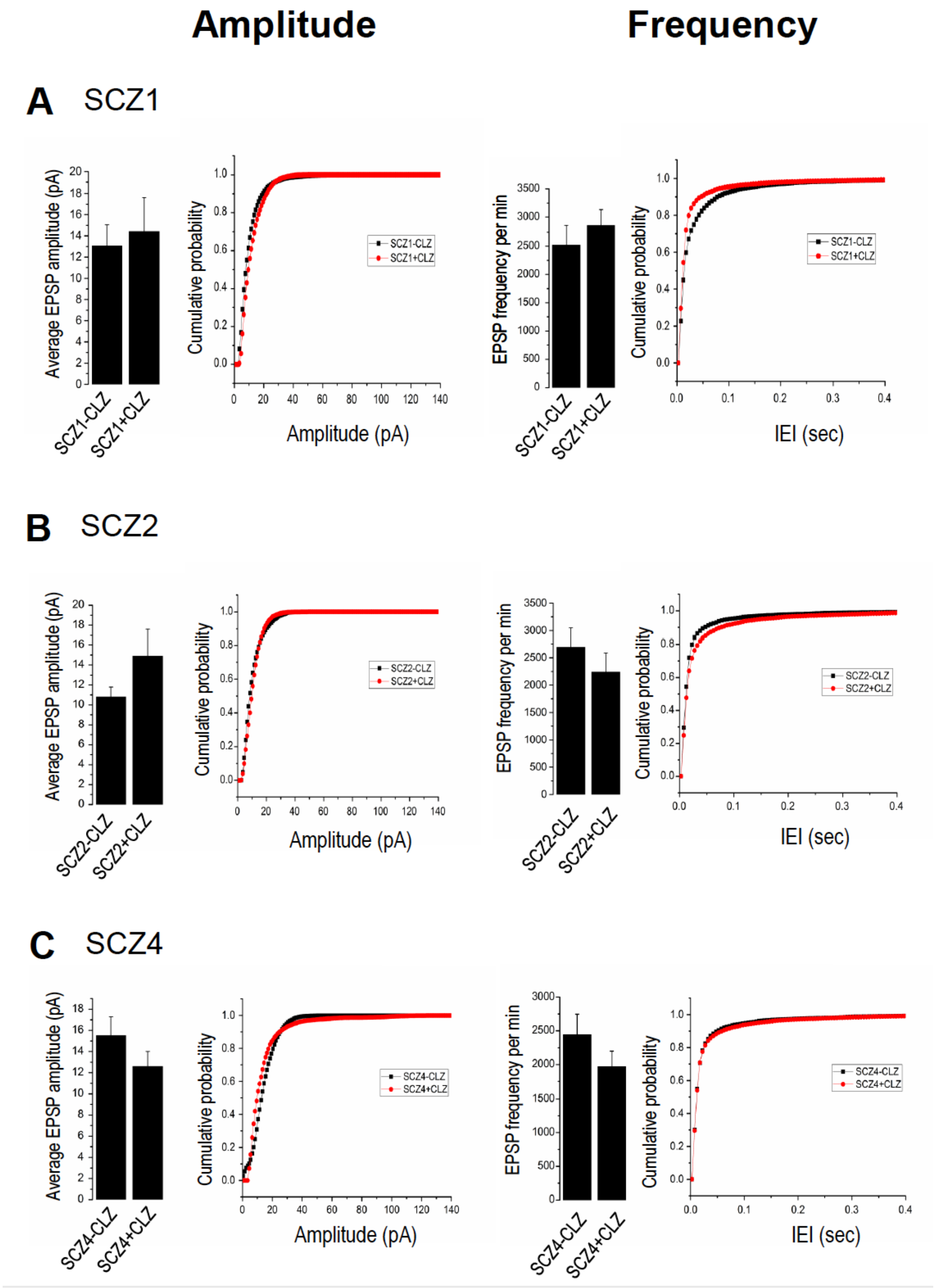

Clozapine shows no statistically significant effect on the activity of NMDA receptors in neurons from clozapine-resistant patients (SCZ1, SCZ2, and SCZ4). Neurons were obtained from cultures at 100-120 days of differentiation that were pre-treated with 1 µM clozapine for 2 weeks prior to experiments. The activity of NMDA receptors was assessed as EPSC amplitudes and frequencies during exposure to 200 µM NMDA. Data were obtained from 15 neurons in each sample and shown as mean ± SEM.

Supplementary Figure 2: Up- and down-regulated genes in clozapine responsive and resistant patients

Bioinformatic analysis of transcriptomic pattern. A: down-regulated genes in cells from clozapine resistant patients when compared with cells from healthy controls. B: down-regulated genes in cells from clozapine responsive patients when compared with cells from healthy controls. C: Up-regulated genes in cells from clozapine resistant patients when compared with cells from clozapine responsive patients.

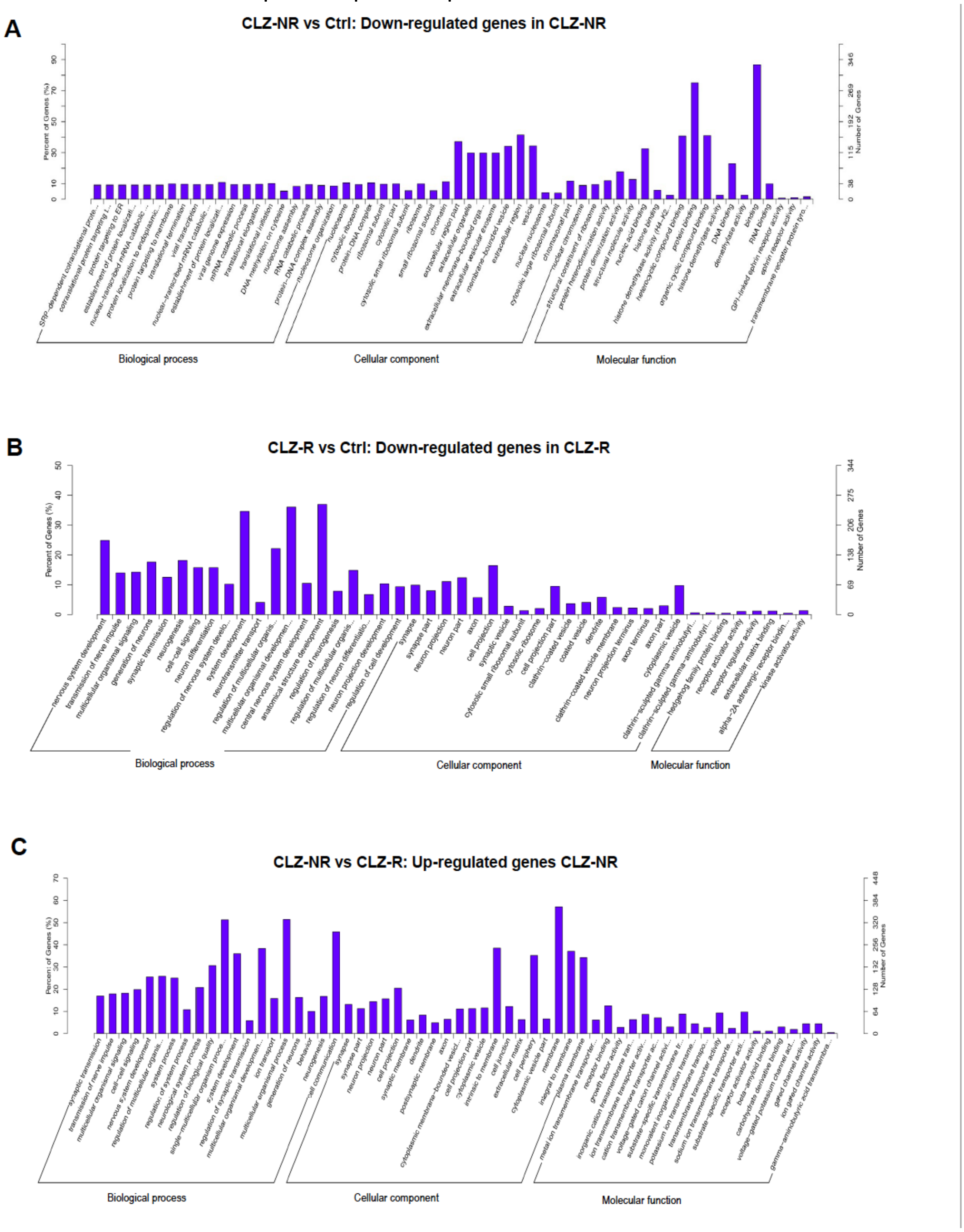

Supplementary Figure 3 (5): Genes upregulated after clozapine pretreatment

Bioinformatic analysis of genes up-regulated in neurons from clozapine responsive patients after the pretreatment with clozapine.

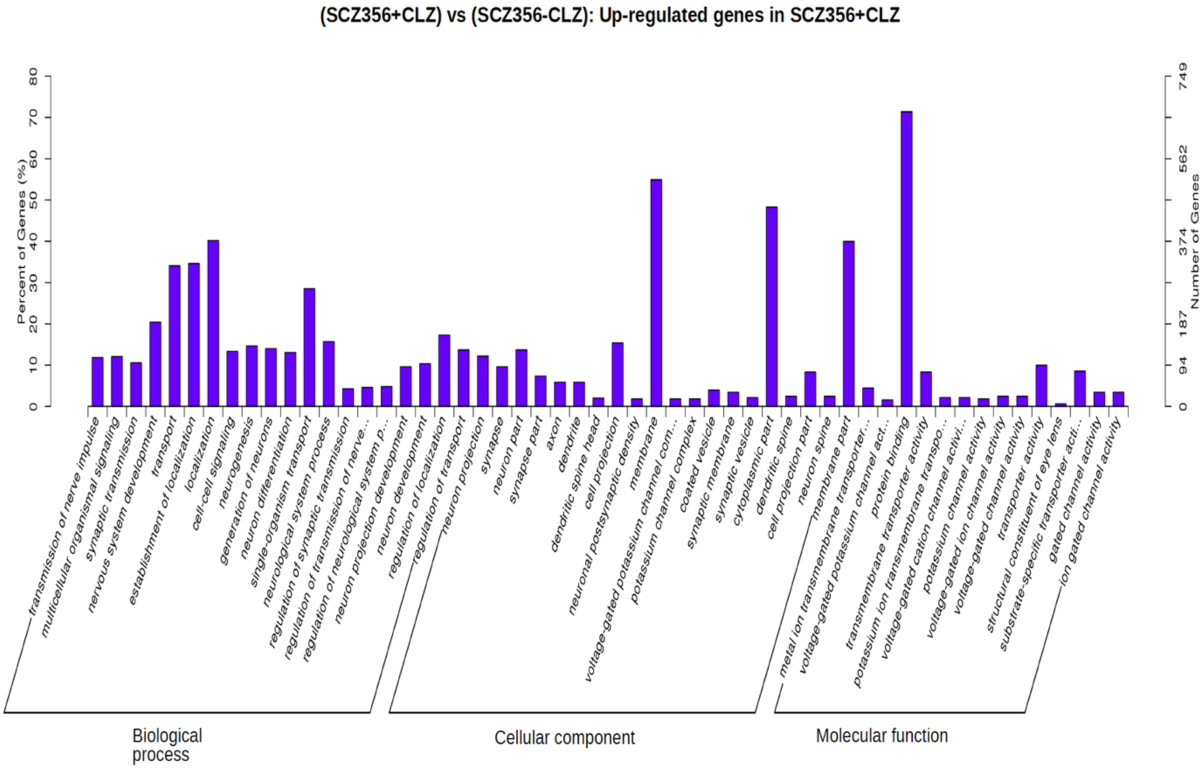

Supplement: Supplementary file 1 [file Data_Sheet_1.PDF]
